# Factors for Returning to Work for Patients with Physical Disabilities and Brain Damage After Industrial Accidents

**DOI:** 10.3390/healthcare14010074

**Published:** 2025-12-27

**Authors:** Dahyeon Koo, Jun Hwa Choi, Eun Suk Choi, Dougho Park

**Affiliations:** 1 Medical Research Institute, Pohang Stroke and Spine Hospital, Pohang 37659, Republic of Korea; 2 Department of Quality Improvement, Pohang Stroke and Spine Hospital, Pohang 37659, Republic of Korea; 3 College of Nursing, Kyungpook National University, Daegu 41566, Republic of Korea; 4 Research Institute of Nursing innovation, Kyungpook National University, Daegu 41566, Republic of Korea; 5 Medical Science and Engineering, School of Convergence Science and Technology, Pohang University of Science and Technology, Pohang 37666, Republic of Korea

**Keywords:** brain injuries, disabled persons, industrial accident, occupational health, return to work

## Abstract

**Background/Objectives**: Return to work (RTW) after an industrial accident is crucial for an individual’s well-being and socioeconomic recovery. This study investigated factors influencing RTW among workers who sustained physical or brain lesion-related disabilities following industrial accidents. **Methods**: Using five-year panel data (2018–2022) from the Panel Study of Workers’ Compensation Insurance of South Korea, we analyzed 340 individuals with physical or brain lesion-related disabilities sustained from industrial accidents. We used logistic regression models to identify factors associated with RTW and return to employed (RTE) status. **Results:** The RTW and non-RTW groups comprised 160 and 180 participants, respectively. Factors associated with non-RTW included female sex (adjusted odds ratio [aOR], 0.33; 95% confidence interval [CI], 0.13–0.86; *p* = 0.023), injury caused by disease (aOR, 0.18; 95% CI, 0.05–0.66; *p* = 0.010), long recovery periods (aOR, 0.27; 95% CI, 0.10–0.72; *p* = 0.009), low self-confidence (aOR, 0.16; 95% CI, 0.07–0.35; *p* < 0.001), and older age (aOR, 0.08; 95% CI, 0.02–0.34; *p* = 0.001). Workers with no blood pressure problems (aOR, 2.21; 95% CI, 1.11–4.38; *p* = 0.024) and longer employment durations (aOR, 3.84; 95% CI, 1.15–12.81; *p* = 0.029) had a higher chance of RTW. Similar factors were associated with RTE, with more emphasis on older age, long recovery periods, low self-confidence, and injury caused by disease. **Conclusions**: Our findings contribute to developing targeted support services and informing policy decisions to improve RTW for workers with physical or brain lesion-related disabilities caused by industrial accidents.

## 1. Introduction

Industrial accidents cause not only physical and psychological suffering for the affected worker but also lead to economic and social losses, creating a significant burden at the national level [1]. According to recent estimates from the International Labour Organization, work-related injuries and diseases account for approximately 3.94% of the global gross domestic product, representing a major economic loss across both developed and developing countries [2]. Furthermore, in cases of severe sequelae such as physical or brain lesion-related disabilities, long-term treatment and rehabilitation are necessary, making return to work (RTW) a challenging process. The proportion of workers with industrial accidents who experience physical disabilities and brain damage annually varies by country and injury type. For example, a meta-analysis reported that approximately 6.3% of work-related injuries result in traumatic brain injury (TBI), and 17.9% of all TBIs are work-related [3]. Regarding physical disability, a Finnish cohort found that after severe occupational injury, 9% of workers remained unable to work (fully or partly) five years post-injury [4]. In Norway, the rate of permanent impairment due to accidents is 800 per 100,000 population, with 400 per 100,000 experiencing disability (restricted activity) [5]. Thus, facilitating RTW after industrial accidents is not only a personal concern but also a critical socioeconomic issue [6].

The importance of return-to-work (RTW) policies for persons with disabilities is also emphasized in international disability frameworks. The Convention on the Rights of Persons with Disabilities (CRPD), ratified by South Korea, highlights in Article 27.1 (k) that States Parties must promote vocational rehabilitation and return-to-work programs for persons with disabilities. Therefore, RTW is not only a matter of individual or workplace benefit but also an internationally recognized obligation for disability-inclusive policy [7].

In South Korea, compensation and rehabilitation for occupational injuries are managed by the Korea Workers’ Compensation and Welfare Service (KCOMWEL) under the Industrial Accident Compensation Insurance Act. The KCOMWEL, together with the Ministry of Employment and Labor, has implemented several programs to promote RTW among industrial accident victims. These include comprehensive rehabilitation initiatives integrating medical treatment, vocational retraining, and job placement services. Regional rehabilitation centers provide individualized case management, occupational therapy, and psychological counseling, while collaborations with employers facilitate gradual work reintegration. Despite these efforts, long-term outcomes remain suboptimal, as workers with physical or brain lesion–related disabilities often require prolonged rehabilitation and experience delayed compensation closure [8].

RTW goes beyond resuming economic activity; it plays a vital role in restoring psychological stability and enhancing self-esteem. RTW provides individuals with financial security and contributes to recovering their quality of life by allowing them to reclaim their role as members of society and affirm their self-worth [9]. Thus, RTW is essential in the recovery process and contributes to improving the individual’s economic and psychological quality of life [10].

RTW is influenced by many factors, including personal characteristics, social aspects, and occupational factors [11,12,13]. However, most studies have focused on industrial accidents in general, and research focusing on the factors affecting RTW in patients with severe sequelae, such as physical or brain lesion-related disabilities, who face enormous challenges in RTW, is lacking. Although several studies have examined RTW after occupational injuries, few have addressed workers with physical or brain lesion–related disabilities, who require prolonged rehabilitation and face unique barriers to reintegration; most Korean studies analyze general injured-worker cohorts, while disability–focused evidence remains limited [14,15].

To address this gap, we analyzed the factors influencing RTW among workers with physical or brain lesion-related disabilities who completed their medical care. By focusing on this specific group, this study aims to provide evidence that may inform the development of policies and social support strategies to enhance RTW.

## 2. Materials and Methods

### 2.1. Data Source and Participants

**Figure 1:**
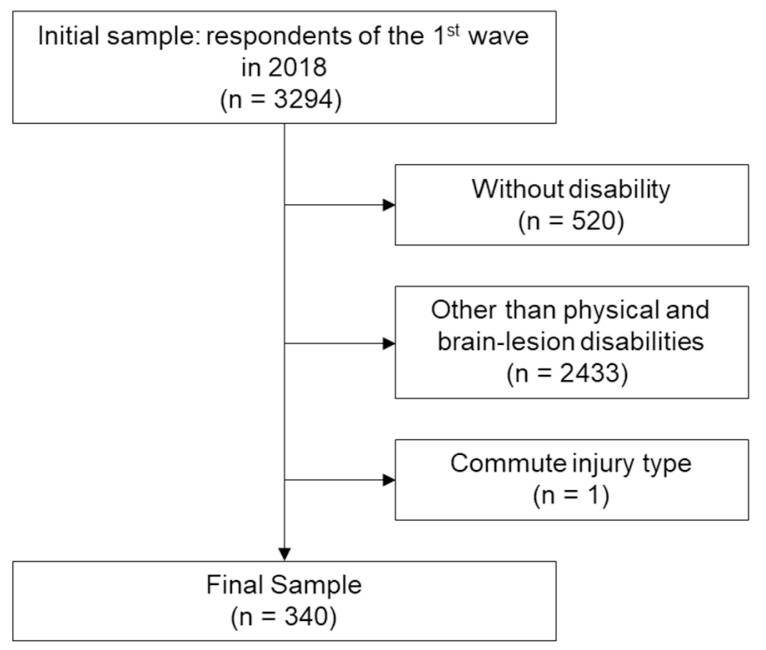
Flowchart illustrating the sample selection process.

This study employed a retrospective longitudinal design using the five-year panel data (2018–2022) from the second cohort of the Panel Study of Workers’ Compensation Insurance (PSWCI), provided by the Labor Welfare Research Institute of the Korea Workers’ Compensation and Welfare Service [16]. The Labor Welfare Research Institute, affiliated with the KCOMWEL, is a government-supported research body under the Ministry of Employment and Labor. It conducts policy research on workers’ compensation, rehabilitation, and return-to-work programs, and manages nationwide data resources, including the PSWCI. We derived the baseline characteristics of the study participants from the results of the first survey conducted in 2018, which included 3294 individuals with physical or brain lesion–related disabilities whose medical care was completed in 2017. We followed them from 2019 to 2022 to observe their RTW status as the outcome. During this process, individuals without a disability rating (n = 520) were excluded, as were those who did not meet the criteria for physical or brain lesion–related disabilities (n = 2433), since the present study specifically targeted individuals with permanent functional impairments requiring long-term rehabilitation and work reintegration support. In addition, one worker with a commuting-related injury was excluded, as commuting accidents, although legally recognized as occupational injuries under the Industrial Accident Compensation Insurance Act, differ from typical work-related accidents in causation, employer involvement, and rehabilitation processes. Ultimately, 340 participants were included in the final analysis (Figure 1).

This study focuses on disabilities resulting from physical injuries or brain lesions caused by industrial accidents, regardless of severity, since the Panel Study of Workers’ Compensation Insurance does not provide a standardized severity classification for physical injuries. Patients with physical or brain lesion–related disabilities were defined according to the official disability classification system of the KCOMWEL, which is based on the Industrial Accident Compensation Insurance Act. In this classification system, physical disabilities correspond to permanent impairments of the locomotor system, including the limbs, spine, or joints, and encompass conditions such as finger or wrist amputation and spinal injuries (e.g., thoracic or lumbar vertebral injuries). In the PSWCI data, these physical disabilities were operationalized as certified disability grades ranging from 1 to 14 (Grade 1 = most severe; Grade 14 = mildest), while brain lesion–related disabilities referred to functional impairments caused by traumatic brain injury or hemorrhagic brain lesions sustained from occupational accidents. Workers without an official disability rating or those with disabilities unrelated to industrial accidents were excluded.

The selection of participants was conducted in accordance with the data provision and usage protocol of the PSWCI. Two researchers independently reviewed the dataset and applied the inclusion and exclusion criteria to ensure accuracy and consistency. The PSWCI cohort was established using a stratified random sampling method by the Labor Welfare Research Institute, selecting 3294 workers who had completed medical care for occupational injuries in 2017 from a total of 75,392 eligible cases. Data were collected through standardized face-to-face interviews by trained field investigators under the supervision of the KCOMWEL. All data provided to the research team were de-identified before analysis.

This study was reviewed and approved by the Institutional Review Board of Pohang Stroke and Spine Hospital (PSSH0475-202410-HR-018-01) and complied with the Declaration of Helsinki. Informed consent was waived due to the study’s retrospective nature and the unidentifiable features of the cohort data by the Institutional Review Board of Pohang Stroke and Spine Hospital.

### 2.2. Outcome Definitions

**Figure 2:**
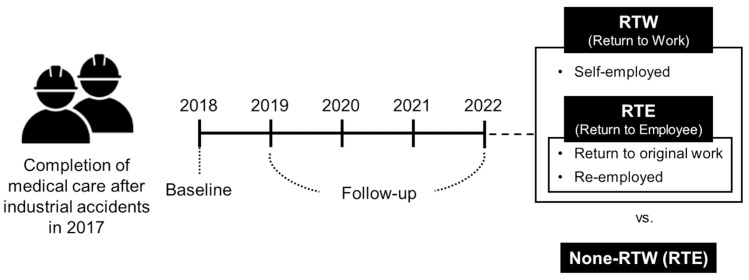
Schematic diagram of the study design and outcome definition.

The dependent variable was the group classification (RTW vs. non-RTW). We defined the RTW group as those who achieved at least one of the following between 2019 and 2022: returning to their original workplace, re-employment, or self-employment. All participants had been employed before the occupational injury. In this study, “work” was defined as any paid employment, including both full-time and part-time positions, regardless of workplace or occupation type. RTW was therefore defined as the resumption of paid work, either at the original workplace, in re-employment, or through self-employment. Individuals who did not achieve RTW during this period were classified in the non-RTW group. For sensitivity analysis, an additional classification, return to employed (RTE), was used to distinguish wage-based employment outcomes from broader work resumption. RTE included participants who returned to their original workplace or were re-employed, while the non-RTE group included all others. This classification was used to differentiate employment-based reintegration from self-employment, which may not necessarily indicate full vocational recovery. Figure 2 presents a schematic diagram of the study design.

A more detailed classification of RTW, such as distinguishing between return to original work with or without task modifications or return to less demanding jobs, was not feasible because the PSWCI dataset does not include information on workplace accommodations or changes in job demands after injury. Therefore, RTW was operationalized as a binary outcome. To partially address this limitation, an additional outcome variable, return to employment (RTE), was used in sensitivity analyses to distinguish wage-based employment from self-employment.

Occupational context was partially considered using the employment status variables available in the PSWCI dataset. Specifically, post-injury economic activity type was classified into categories such as return to the original workplace, re-employment, self-employment, unemployment, and economically inactive status. These categories reflect different employment contexts following injury and were used as proxies for occupational characteristics. However, the PSWCI does not provide detailed information on specific job titles, managerial responsibilities, or task-level physical demands. Therefore, it was not possible to directly distinguish between managerial, service-oriented, or physically demanding occupations.

### 2.3. Covariates

Table S1 defines the study variables, including RTW status, demographic factors (sex, age group, years of education, and marital status), health and disability-related factors (disability rating and chronic illnesses, such as diabetes and blood pressure problems), rehabilitation service utilization (use of occupational and social rehabilitation services), industrial accident and work-related factors (duration of employment, type of work-related injury, and recovery period), and psychological factors (self-confidence). In this study, blood pressure problems were defined as self-reported physician-diagnosed hypertension, and self-confidence was measured using a self-rated five-point Likert scale assessing the individual’s perceived confidence in returning to work. For analysis, self-confidence responses were categorized into three groups: negative (scores 1–2), neutral (score 3), and positive (scores 4–5).

Rehabilitation service utilization was defined as participation in at least one rehabilitation program provided by the Korea Workers’ Compensation and Welfare Service after completion of medical care for the occupational injury. Based on administrative data linked to the PSWCI (as of November 2022), this variable indicated whether participants had ever used any occupational rehabilitation service (e.g., job training, work ability enhancement, job placement support, or employment counseling) or social rehabilitation service (e.g., psychological counseling, mentoring, or rehabilitation sports) regardless of the duration or frequency of participation.

The type of work-related injury was classified based on administrative records provided by the KCOMWEL. According to the Industrial Accident Compensation Insurance system, occupational injuries are categorized into work-related accidents, work-related diseases, and commuting accidents. Work-related disease refers to any illness or disorder officially recognized as caused or aggravated by occupational factors, including musculoskeletal disorders, noise-induced hearing loss, and diseases related to chemical or physical exposure.

All participants in the PSWCI cohort were covered under the same national workers’ compensation system regulated by the Industrial Accident Compensation Insurance Act in Korea. Therefore, differences in workplace injury policies or sick leave systems were not considered as covariates, since institutional conditions and compensation procedures are uniformly applied across all insured workers.

### 2.4. Statistical Analysis

All statistical analyses were performed using R software version 4.3.1 (R Core Team, R Foundation for Statistical Computing, Vienna, Austria, https://www.R-project.org/). We established a multivariable logistic regression model, and the results were presented as adjusted odds ratios (aORs) with 95% confidence intervals (CIs). To select the optimal model, we performed backward stepwise elimination and assessed model fit based on the lowest Akaike information criterion value. We calculated variance inflation factors (VIFs) to evaluate multicollinearity among independent variables. All VIF values were <10, indicating no multicollinearity issues. Missing data were handled using complete case analysis. Further, possible interaction effects between disability grade and rehabilitation service utilization (both occupational and social) were analyzed to evaluate whether the association between rehabilitation participation and return-to-work outcomes differed according to the severity of disability. Interaction terms (disability grade × occupational rehabilitation service and disability grade × social rehabilitation service) were tested using likelihood ratio tests in logistic regression models. However, none of the interaction effects were statistically significant (all *p* > 0.05). Therefore, only main effects were retained in the final model for parsimony and interpretability.

## 3. Results

### 3.1. Comparative Baseline Characteristics of RTW and Non-RTW Groups

There were 160 and 180 participants in the RTW and non-RTW groups, respectively. Baseline characteristics between the RTW and non-RTW groups were compared using the chi-square test. The proportion of individuals aged < 60 years was relatively higher in the RTW group than in the non-RTW group, while the proportion of individuals aged ≥ 60 years was significantly higher in the non-RTW group than in the RTW group (62.2% vs. 35.0%; *p* < 0.001). The utilization of occupational rehabilitation services in the RTW group was significantly higher than in the non-RTW group (28.1% vs. 17.8%; *p* = 0.032). Regarding the type of work-related injury, the RTW group had a significantly higher proportion of physical injuries than the non-RTW group (96.9% vs. 85.5%; *p* = 0.001). Concerning health-related factors, diabetes prevalence did not differ significantly between the groups (*p* = 0.102), but the prevalence of blood pressure problems was significantly lower in the RTW group than in the non-RTW group (20.6% vs. 35.5%; *p* = 0.005) (Table 1).

There were 145 and 195 participants in the RTE and non-RTE groups, respectively. Table S2 presents the baseline characteristics of these groups.

### 3.2. Factors Influencing RTW

Logistic regression analysis revealed several variables that significantly influenced the likelihood of RTW. Females were less likely to RTW than males (aOR, 0.33; 95% CI, 0.13–0.86; *p* = 0.023). Workers without blood pressure problems were significantly more likely to RTW compared with those without these conditions (aOR, 2.21; 95% CI, 1.11–4.38; *p* = 0.024). Regarding the type of industrial accident, workers whose injuries were caused by disease were significantly less likely to RTW than those who were physically injured (aOR, 0.18; 95% CI, 0.05–0.66; *p* = 0.010). Workers with a recovery period > 24 months were significantly less likely to RTW than those with a recovery period ≤ 6 months (aOR, 0.27; 95% CI, 0.10–0.72; *p* = 0.009). Workers with low self-confidence had a significantly lower likelihood of returning to work than those with a neutral attitude (aOR, 0.16; 95% CI, 0.07–0.35; *p* < 0.001). Finally, participants with an employment duration of 5–9 years were significantly more likely to RTW compared with those with an employment duration < 1 year (aOR, 3.84; 95% CI, 1.15–12.81; *p* = 0.028) (Table 2).

We also performed a sensitivity analysis using the RTE group, which included only those who had returned to their original workplace or were re-employed. The logistic regression analysis results for the RTE group revealed that the likelihood of returning to work was significantly lower for workers whose injuries were caused by disease than by physical injury (aOR, 0.17; 95% CI, 0.04–0.67; *p* = 0.011), those whose recovery was >24 months compared with a recovery ≤ 6 months (aOR, 0.24; 95% CI, 0.09–0.63; *p* = 0.004), those with low self-confidence compared with neutral self-confidence (aOR, 0.15; 95% CI, 0.07–0.33; *p* < 0.001), and those aged ≥ 60 years (aOR, 0.08; 95% CI, 0.02–0.33; *p* < 0.001) (Table S3). Thus, when analyzed separately, four variables (injuries caused by disease, recovery > 24 months, low self-confidence, and an age ≥ 60 years) were identified as significant factors common to both the RTW and RTE groups.

## 4. Discussion

This study examined factors affecting RTW among workers with physical or brain lesion–related disabilities. Physical or brain lesion-related disabilities are sequelae of industrial accidents that greatly limit the daily life and work activities of affected individuals. Workers with these conditions face considerable challenges, such as physical impairment, cognitive decline, and mental health struggles, making RTW difficult [13,14]. Thus, understanding the RTW process in this population and providing tailored support are essential [15]. Physical or brain lesion-related disabilities are severe sequelae of industrial accidents that greatly limit the daily life and work activities of affected individuals. Workers with these conditions face considerable challenges, such as physical impairment, cognitive decline, and mental health struggles, making RTW difficult [16,17]. Thus, understanding the RTW process in this population and providing tailored support are essential [18]. We identified four key factors as common to the RTW and RTE groups: type of industrial accident, recovery period, self-confidence, and age group.

A previous study using workers’ compensation panel data identified age and sex as significant factors for RTW, with the recovery period and rehabilitation service use also influencing the likelihood of returning to work [18]. In addition, broader studies of occupational injuries have primarily focused on injured workers in general or on specific body regions, such as hand or upper limb injuries, and have reported that factors like age, sex, years of education, and injury type significantly influence RTW outcomes. However, most of these studies were limited to workers with localized musculoskeletal injuries and rarely included those with severe or permanent disabilities, such as brain lesions or major physical impairments [19]. In contrast, our study specifically examined workers with physical or brain lesion–related disabilities, who face more complex challenges in work reintegration. The key factors associated with RTW in this population included sex, chronic diseases, type of industrial accident, recovery period, self-confidence, age group, and employment duration. Older workers and those with extended recovery periods were less likely to RTW, which aligns with previous findings [20]. The presence of cognitive impairment and persistent functional limitations likely makes early reintegration more challenging, underscoring the need for comprehensive rehabilitation approaches integrating medical, vocational, and psychological support.

Logistic regression analyses of the RTW and RTE groups aimed to provide more detailed information on returning to the original workplace and to examine various factors influencing RTW from multiple perspectives. In the RTW analysis, sex and blood pressure problems were identified as additional significant factors to those determined as significant in both the RTE and RTW analyses (i.e., injury type, recovery period, self-confidence, and age group). Meanwhile, in the RTE analysis, low self-confidence and injuries caused by disease were more prominently highlighted. Specifically, injuries caused by disease, prolonged recovery periods, low self-confidence, and older age were major factors reducing the likelihood of returning to work.

Based on these key findings, we propose targeted strategies corresponding to each determinant identified in the analysis. First, for workers with injuries caused by disease, medical services should incorporate disease-specific rehabilitation protocols and long-term follow-up systems, as this group showed a significantly lower likelihood of RTW compared with those with physical injuries. Second, to address the challenges associated with prolonged recovery periods, stepwise RTW programs and continuous psychological support should be provided, particularly for individuals requiring extended medical or functional rehabilitation. Third, because low self-confidence emerged as a strong psychological barrier to RTW, workplace mentoring and counseling programs should be strengthened to help workers rebuild confidence and facilitate reintegration. Finally, given that older age was consistently associated with a lower likelihood of RTW, tailored job placement and retraining programs should be developed for older workers to accommodate their physical and cognitive limitations. By directly linking these recommendations to the corresponding results, our findings provide an evidence-based framework for developing comprehensive RTW strategies tailored to workers with physical or brain lesion–related disabilities.

In terms of policy implications, our results provide empirical evidence supporting the expansion of rehabilitation service eligibility and funding for long-term or disease-related injury cases. These strategies should be implemented through collaboration between medical institutions, the KCOMWEL, and employers, ultimately contributing to both clinical and policy improvements in work reintegration.

Future research should explore longitudinal changes in RTW trajectories to understand long-term adaptation and the sustainability of employment. Additionally, qualitative studies examining workers’ subjective experiences could provide deeper insight into psychosocial barriers and facilitators. Linking medical records with compensation data may further clarify the role of clinical severity and rehabilitation intensity in RTW outcomes.

This study has several limitations. First, due to its retrospective design and observational nature, it was difficult to draw causal inferences about the factors influencing RTW from our findings. Second, although employment context after injury was partially considered using economic activity type, detailed information on job responsibilities or physical task demands (e.g., managerial versus service-oriented work) was unavailable in the workers’ compensation panel data, which may have influenced RTW outcomes. Third, although a more granular classification of RTW would be desirable, information on workplace modifications or changes in job demands after injury was unavailable in the PSWCI dataset, limiting the ability to classify RTW into more detailed categories. Fourth, because the workers’ compensation panel data used in this study did not include detailed clinical information at the time of injury, it was difficult to determine the initial condition of the patients or differences in their rehabilitation processes. Fifth, due to the cohort design, the analysis was based on the first RTW event during the 5-year follow-up period, limiting the ability to capture sequential changes or long-term trends in RTW.

## 5. Conclusions

This study analyzed the factors influencing the RTW of workers with physical or brain lesion-related disabilities after industrial accidents. Injury type, recovery period, self-confidence, and age group were identified as key factors. Our results provide a foundation for supporting the successful RTW of workers with physical or brain lesion-related disabilities. Furthermore, our findings contribute to developing tailored unmet medical services and informing future policy initiatives.

## Figures and Tables

**Table 1:** Baseline characteristics.

Variables	RTW Group (0 = 160)	Non-RTW Group (n = 180)	*p*-Value
RTW state, n (%)			<0.001
Return to original workplace	41 (25.2)	0 (0.0)	
Re-employed	104 (65.0)	0 (0.0)	
Self-employed	15 (9.4)	0 (0.0)	
Non-RTW	0 (0.0)	180 (100.0)	
Male, n (%)	147 (91.9)	158 (87.8)	0.288
Age group, n (%)			<0.001
<40 years	20 (12.5)	6 (3.3)	
40–49 years	32 (20.0)	19 (10.6)	
50–59 years	52 (32.5)	43 (23.9)	
≥60 years	56 (35.0)	112 (62.2)	
Years of education, n (%)			0.093
≤6 years	27 (16.9)	43 (23.9)	
7–12 years	101 (63.1)	114 (63.3)	
>12 years	32 (20.0)	23 (12.8)	
Disability rating, n (%)			<0.001
Severe (1–7)	76 (47.5)	124 (68.9)	
Mild–moderate (8–14)	84 (52.5)	56 (31.1)	
Occupational rehabilitation service, n (%)			0.032
Not used	115 (71.9)	148 (82.2)	
Used	45 (28.1)	32 (17.8)	
Social rehabilitation service, n (%)			>0.999
Not used	107 (66.9)	120 (66.7)	
Used	53 (33.1)	60 (33.3)	
Duration of employment, n (%)			0.259
<1 year	90 (56.2)	118 (65.6)	
1–4 years	45 (28.1)	40 (22.2)	
5–9 years	16 (10.0)	11 (6.1)	
≥10 years	9 (5.6)	11 (6.1)	
Type of work-related injury, n (%)			0.001
Physical injury	155 (96.9)	153 (85.5)	
Disease	5 (3.1)	26 (14.5)	
Recovery period, n (%)			<0.001
≤6 months	43 (26.9)	25 (13.9)	
7–24 months	97 (60.6)	75 (41.7)	
>24 months	20 (12.5)	80 (44.4)	
Marital status, n (%)			0.833
Not married	20 (12.5)	26 (14.4)	
Married	112 (70.0)	121 (67.2)	
Other	28 (17.5)	33 (18.3)	
Type of disability, n (%)			<0.001
Physical disability	157 (98.1)	150 (83.3)	
Brain lesion-related disability	3 (1.9)	30 (16.7)	
Diabetes, n (%)	22 (13.8)	38 (21.1)	0.102
Blood pressure problems, n (%)	33 (20.6)	63 (35.0)	0.005
Self-confidence, n (%)			<0.001
Low	14 (8.8)	91 (50.6)	
Neutral	60 (37.5)	47 (26.1)	
High	86 (53.8)	42 (23.3)	

Abbreviations: RTW, return to work.

**Table 2:** Results of logistic regression analysis for return to work after work-related injuries.

Variables	aOR	95% CI	*p*-Value
Female	0.33	0.13–0.86	0.023
Age group			
<40 years	Reference		
40–49 years	0.54	0.12–2.43	0.422
50–59 years	0.28	0.07–1.18	0.083
≥60 years	0.08	0.02–0.34	0.001
Years of education			
≤6 years	Reference		
7–12 years	0.64	0.30–1.36	0.241
>12 years	0.87	0.30–2.55	0.796
Disability rating			
Severe (1–7)	Reference		
Mild–moderate (8–14)	1.71	0.87–3.36	0.118
Use of occupational rehabilitation service	1.32	0.66–2.64	0.431
Use of social rehabilitation service	1.01	0.52–1.97	0.975
Duration of employment			
<1 year	Reference		
1–4 years	1.55	0.75–3.22	0.241
5–9 years	3.84	1.15–12.81	0.028
≥10 years	2.28	0.61–8.46	0.218
Type of work-related injury			
Physical injury	Reference		
Disease	0.18	0.05–0.66	0.010
Recovery period			
≤6 months	Reference		
7–24 months	0.81	0.39–1.70	0.579
>24 months	0.27	0.10–0.72	0.009
Marital status			
Not married	Reference		
Married	2.48	0.88–7.01	0.087
Other	2.77	0.83–9.24	0.099
Without diabetes	0.96	0.42–2.17	0.918
Without blood pressure problems	2.21	1.11–4.38	0.024
Self-confidence			
Neutral	Reference		
Low	0.16	0.07–0.35	<0.001
High	1.65	0.87–3.12	0.124

Abbreviations: aOR, adjusted odds ratio; CI, confidence interval.

## Data Availability

Data were obtained from the Korea Workers′ Compensation & Welfare Service and are available at https://www.pswci.or.kr/Data/Download.aspx?rnd=672818.13671478 (accessed on 31 October 2024) with the permission of the Korea Workers′ Compensation & Welfare Service.

## Supplementary Materials

The following supporting information can be downloaded at https://www.mdpi.com/article/10.3390/healthcare14010074/s1, Table S1. Variable categories; Table S2. Baseline characteristics when the return to work is defined as returning to the original work and being re-employed; Table S3. Logistic regression model for returning to original work and being re-employed.

## Abbreviations

RTW	Return to work
TBI	Traumatic brain injury
KCOMWEL	Korea Workers’ Compensation and Welfare Service
PSWCI	Panel Study of Workers’ Compensation Insurance
RTE	Return to employed
aOR	Adjusted odds ratio
CI	Confidence interval
VIF	Variance inflation factor
