# Supplementary material for: Factors for Returning to Work for Patients with Physical Disabilities and Brain Damage After Industrial Accidents
Source: Healthcare (Basel). 2025 Dec 27;14(1):74. doi: 10.3390/healthcare14010074 (PMC12785851; doi:10.3390/healthcare14010074)
Supplement: Supplementary file 1 [file healthcare-14-00074-s001.zip › healthcare-4007973-supplementary.pdf]

Supplementary Tables

Table S1. Variable categories.

Table S2. Baseline characteristics when the return to work is defined as returning to the original work and being re-employed.

Table S3. Logistic regression model for returning to original work and being re-employed.

Table S1. Variable categories.

Variables	Categories
Return to work state	Return to original work
	Re-employed
	Self-employed
	Non-return to work
Sex	Male
	Female
Age group	<40 years
	40–49 years
	50–59 years
	≥60 years
Education level	≤6 years
	7–12 years
	>12 years
Disability rating	Severe (1–7)
	Mild–moderate (8–14)
Occupational rehabilitation service	Not used
	Used
Social rehabilitation service	Not used
	Used
Duration of employment	<1 year
	1–4 years
	5–9 years
	≥10 years
Type of work-related injury	Physical injury
	Disease
Recovery period	≤6 months
	7–24 months
	>24 months
Marital status	Not-married
	Married
	Other
Type of disability	Physical disability
	Brain lesion-related disability
Diabetes	-
Blood pressure problems	-
Self-confidence	Low
	Neutral
	High

Table S2. Baseline characteristics when the return to work is defined as returning to the original work and being re-employed.

Variables	RTE group (n = 145)	non-RTE group (n = 195)	*p*-value
RTW state, n (%)			<0.001
Return to original work	41 (28.3)	0 (0.0)	
Re-employed	104 (71.7)	0 (0.0)	
Self-employed	0 (0.0)	15 (7.7)	
Non-RTW	0 (0.0)	180 (92.3)	
Male, n (%)	132 (91.0)	173 (88.7)	0.607
Age group, n (%)			<0.001
<40 years	19 (13.1)	7 (3.6)	
40–49 years	28 (19.3)	23 (11.8)	
50–59 years	47 (32.4)	48 (24.6)	
≥60 years	51 (35.2)	117 (60.0)	
Education level, n (%)			0.406
≤6 years	26 (17.9)	44 (22.6)	
7–12 years	92 (63.4)	123 (63.1)	
>12 years	27 (18.6)	28 (14.4)	
Disability rating, n (%)			0.001
Severe (1–7)	70 (48.3)	130 (66.7)	
Mild–moderate (8–14)	75 (51.7)	65 (33.3)	
Occupational rehabilitation service, n (%)			0.138
Not-used	106 (73.1)	157 (80.5)	
Used	39 (26.9)	38 (19.5)	
Social rehabilitation service, n (%)			0.872
Not-used	98 (67.6)	129 (66.2)	
Used	47 (32.4)	66 (33.8)	
Duration of employment, n (%)			0.357
<1 year	81 (55.9)	127 (65.1)	
1–4 years	41 (28.3)	44 (22.6)	
5–9 years	14 (9.7)	13 (6.7)	
≥10 years	9 (6.2)	11 (5.6)	
Type of work-related injury, n (%)			0.001
Physical injury	141 (97.2)	167 (86.1)	
Disease	4 (2.8)	27 (13.9)	
Recovery period, n (%)			<0.001
≤6 months	38 (26.2)	30 (15.4)	
7–24 months	92 (63.4)	80 (41.0)	
>24 months	15 (10.3)	85 (43.6)	

Marital status, n (%)			0.859
Not-married	18 (12.4)	28 (14.4)	
Married	100 (69.0)	133 (68.2)	
Other	27 (18.6)	34 (17.4)	
Type of disability, n (%)			<0.001
Physical disability	142 (97.9)	165 (84.6)	
Brain lesion-related disability	3 (2.1)	30 (15.4)	
Diabetes, n (%)	19 (13.1)	41 (21.0)	0.080
Blood pressure problems, n (%)	113 (77.9)	131 (67.2)	0.040
Self-confidence, n (%)			<0.001
Low	58 (40.0)	49 (25.1)	
Neutral	13 (9.0)	92 (47.2)	
High	74 (51.0)	54 (27.7)	

Abbreviation: RTE, return to being employment.

Table S3. Logistic regression model for returning to original work and being re-employed.

Variables	adjusted OR	95% CI	*p*-value
Female	0.47	0.19-1.17	0.104
Age group			
<40 years	Reference		
40–49 years	0.38	0.10-1.50	0.167
50–59 years	0.28	0.07-1.05	0.060
≥60 years	0.08	0.02-0.33	<0.001
Education level			
≤6 years	Reference		
7–12 years	0.61	0.29-1.27	0.189
>12 years	0.60	0.21-1.68	0.328
Disability rating			
Severe (1–7)	Reference		
Mild–moderate (8–14)	1.20	0.64-2.24	0.572
Use of occupational rehabilitation service	1.03	0.53-1.99	0.931
Use of social rehabilitation service	0.95	0.51-1.78	0.872
Duration of employment			
<1 year	Reference		
1–4 years	1.40	0.71-2.78	0.335
5–9 years	2.85	0.93-8.69	0.066
≥10 years	2.89	0.81-10.39	0.103
Type of work-related injury			
Physical injury	Reference		
Disease	0.17	0.04-0.67	0.011
Recovery period			
≤6 months	Reference		
7–24 months	1.01	0.51-2.02	0.970
>24 months	0.24	0.09-0.63	0.004
Marital status			
Not-married	Reference		
Married	1.96	0.73-5.28	0.185
Other	2.51	0.79-8.01	0.119
Without diabetes	1.20	0.54-2.65	0.654
Without blood pressure problems	1.54	0.80-2.94	0.195
Self-confidence			
Neutral	Reference		

Low	0.15	0.07-0.33	<0.001
High	1.06	0.58-1.94	0.844

Abbreviations: OR, odds ratio; CI, confidence interval.
